# An In Vitro and In Silico Study of Luteolin-Loaded Zinc Oxide Nanoparticles: Enhancing Bioactivity and Efficacy for Advanced Therapeutic Applications Against Cariogenic Microorganisms

**DOI:** 10.7759/cureus.68058

**Published:** 2024-08-28

**Authors:** Kethan Umakanth, Taniya Mary Martin, Meenakshi Sundaram K

**Affiliations:** 1 Zebra Fish Facility, Department of Anatomy, Saveetha Dental College and Hospitals, Saveetha Institute of Medical and Technical Sciences (SIMATS), Saveetha University, Chennai, IND

**Keywords:** glucosyltransferases, fourier transform infrared spectroscopy (ftir), molecular docking, dental biofilm, cariogenic microorganisms, antimicrobial activity in natural products, lavender-extracted nanoparticles

## Abstract

Introduction

Recent studies have explored alternative methods to enhance caries prevention and treatment. Luteolin compound has been noted for its antimicrobial properties, while zinc nanoparticles (Zn NPs) are recognized for their potent antibacterial effects. This study investigates the synthesis, characterization, and antimicrobial efficacy of luteolin-loaded Zn oxide NPs (Luteo-ZnONPs) against cariogenic bacteria. By combining the biofilm-targeting capabilities of luteolin with the antimicrobial properties of Zn NPs, we aim to explore a novel approach for dental caries management.

Methods

Luteo-ZnONPs were synthesized and characterized using ultraviolet-visible (UV-vis) and Fourier transform infrared (FTIR) spectroscopy, confirming their successful formation and stability. Antimicrobial efficacy was assessed through minimum inhibitory concentration (MIC), demonstrating effectiveness against cariogenic bacteria such as *Escherichia coli, Enterococcus faecalis, Pseudomonas aeruginosa*, and *Streptococcus mutans* in different concentrations. The agar well plate method was employed to analyze the growth inhibitory effect of Luteo-ZnONPs (50 and 100 µg/ml, respectively). Streptomycin (100 µg/ml) was used as a positive control. The results (zone of inhibition (ZOI) in millimeter, mm) were represented as mean ± standard deviation. One-way analysis of variance (ANOVA) was employed to detect the significance (p < 0.05) between the groups. Cytotoxicity was analyzed using the 3-(4,5-dimethylthiazol-2-yl)-2,5-diphenyltetrazolium bromide (MTT) assay against MG63 cells, and doxorubicin was used as a positive control. Wilcoxon rank test was used for the statistical method. Gyrase B was downloaded from Protein Data Bank (PDB id: 6F86) and docked against luteolin using Autodock software (version 4.2). The binding score was presented as kcal/mol in table format.

Results

Characterization results showed that UV-vis spectroscopy revealed characteristic peaks, indicating the successful synthesis and stability of Luteo-ZnONPs. FTIR spectroscopy confirmed the presence of functional groups from luteolin compound interacting with the Zn NPs. It showed effective inhibition against *E. coli *on 50 µg/ml as 12.45 mm as ZOI and increased with concentration (100 µg/ml as 17.13 mm). It showed minimal ZOI on *E. faecalis* (8.12, 12.21 on 50 and 100µg/ml, respectively). The cytotoxicity of Luteo-ZnONPs was lesser than doxorubicin on MG63 cells with statistical high significance (p < 0.0014). These results showed that Luteo-ZnONPs had effective antimicrobial nature against *Enterococcus *family. Thus, gyrase B from *E. coli *was selected for the molecular docking analysis. The catalytic tunnel in gyrase B *(E. coli*, PDB: 6F86), influenced by Luteo-ZnONPs, indicated potential for novel, broad-spectrum antimicrobials via selective inhibition at conserved active sites.

Conclusion

The agar well plate and MIC confirmed that Luteo-ZnONPs exhibited potent antibacterial activity, especially at higher concentrations compared to streptomycin. One- way ANOVA demonstrated significant differences in antibacterial efficacy between treatments, validating its superior performance. Its strong interaction on in silico* *level showed the targeted mechanism of action. Luteo-ZnONPs showed lesser toxicity than doxorubicin on MG63 cells. These findings underscore the potential of its broad spectrum antimicrobial nature paving the way for its development into innovative, nontoxic therapeutic solutions.

## Introduction

Dental caries, commonly known as tooth decay, is one of the most prevalent chronic diseases worldwide, affecting individuals of all ages. This multifactorial disease results from the demineralization of tooth enamel due to acid production by cariogenic microorganisms. These microorganisms colonize the oral cavity, forming biofilms on tooth surfaces. Biofilms are complex communities of bacteria and other microorganisms that protect the bacteria from environmental stresses and antimicrobial agents, making them challenging to eradicate. This leads to the gradual breakdown of tooth enamel and dentin, resulting in cavities and potentially more severe dental issues if left untreated [1-3]. The primary culprits behind dental caries include a variety of microorganisms such as *Streptococcus mutans *(*S. mutans*)*, Enterococcus* *faecalis *(*E. faecalis*)*, Escherichia coli *(*E. coli*), and *Pseudomonas aeruginosa *(*P. aeruginosa*). *P. aeruginosa, *a versatile pathogen, is often found in the biofilms forming on the teeth of individuals suffering from severe dental caries. This condition, characterized by rampant tooth decay, represents a significant global public health issue. The presence of *P. aeruginosa *in dental plague exacerbates the progression of carries by enhancing biofilm resilience and contributing to the acidic environment that promotes enamel demineralization. Understanding the role of *P. aeruginosa *in these biofilms is crucial for developing targeted strategies to prevent and treat severe dental caries, aiming to mitigate its impact on affected populations. *S. mutans *is another major contributor to dental caries. This bacterium is particularly adept at adhering to the tooth surface and producing glucans from dietary sugars, which facilitate the formation of a robust biofilm. When exposed to fermentable carbohydrates like sucrose and fructose, *S. mutans *metabolizes these sugars , producing acids as byproducts. These acids lower the pH in the oral environment, leading to the demineralization of tooth enamel and the formation of the cavities [4-7]. The ability of *S. mutans *to thrive in such environments and form resilient biofilms makes it a primary contributor to tooth decay, underscoring the importance of controlling dietary sugar intake and maintaining oral hygiene to prevent caries. *E. faecalis *is another bacterium associated with dental caries, particularly in the context of endodontic failure. This species exhibits high resistance to common disinfection agents, leading to persistent intra-radicular or extra-radicular infections. *E. faecalis *can survive in harsh conditions, including nutrient-depleted environments, and often forms resilient biofilms that protect it from antimicrobial treatments. This persistence contributes to the failure of root canal therapy, necessitating advanced and targeted disinfection strategies to effectively eradicate these resilient bacterial populations and ensure successful endodontic outcomes. *Escherichia coli, *typically associated with fecal contamination, is not a resident but rather a transient member of the oral microbiota. Its occasional presence in the oral cavity may indicate environmental exposure to contaminated sources. The isolation of *E.coli *in the oral cavity raises concerns about potential fecal-oral transmission routes for pathogenic organisms [5-8]. This transient colonization underscores the importance of maintaining oral hygiene and preventing oral ingestion of pathogens that could lead to gastrointestinal infections. Monitoring and preventing the presence of*E. coli *in the oral environment* *are crucial for mitigating health risks associated with microbial contamination. *Staphylococcus aureus *(*S. aureus*),* *traditionally associated with skin and nasal carriage, can transiently colonize the oral cavity through various environmental exposures. Its presence in the oral microbiota suggests a potential influence on oral health, yet their specific contributions, whether beneficial, neutral, or potentially harmful, are still not fully understood. While some studies suggest that *Staphylococci *can contribute to oral infections, others indicate a more commensal relationship within the oral flora. The role of *S. aureus* in oral health remains a subject of debate and ongoing research. Given the complex nature of dental biofilms and the resilience of cariogenic microorganisms, innovative approaches to prevent and treat dental caries are necessary [8-12]. By including them, the scope of the present antimicrobial testing is expanded to encompass more clinical circumstances in dentistry than merely the development of caries. Traditional treatments often fall short in effectively targeting biofilms and are increasingly limited by the growing issue of antibiotic resistance [12-13]. As a result, there is a need for novel antimicrobial agents that can disrupt biofilms and inhibit the growth of cariogenic microorganisms. In recent years, nanoparticles (NPs) have gained significant attention for their potent antimicrobial properties. Among these, zinc (Zn) NPs have been extensively studied for their broad-spectrum antimicrobial effects. Zn NPs are effective against a wide range of microorganisms, including bacteria, fungi, and viruses. Their antimicrobial action is primarily attributed to their ability to disrupt microbial cell membranes, generate reactive oxygen species and interfere with microbial DNA and protein functions. These mechanisms collectively contribute to the bacterial effects of silver nanoparticles (AgNPs), making them a promising candidate for combating dental caries. In this study, however, we explore the potential of luteolin-loaded zinc oxide nanoparticles (Luteo-ZnONPs) as an curing agent in oral health [1,14-16].

Luteolin, known for its antimicrobial properties combined with Zn, an essential trace element known for its antibacterial effects, offers a novel approach to caries prevention and treatment. Luteo-ZnONPs were synthesized and characterized using ultraviolet-visible (UV-vis) spectroscopy and Fourier transform infrared (FTIR) spectroscopy to confirm their size, shape, and stability. The synthesis process involves extracting essential oils from luteolin compound and incorporating Zn ions to form NPs [1-6]. The antibacterial efficacy of Luteo-ZnONPs was assessed through various assays, which demonstrated significant inhibition of cariogenic bacteria. To understand the interaction between Luteo-ZnONPs and bacterial enzymes involved in biofilm formation, molecular docking studies were performed. These studies revealed strong binding affinities of Luteo-ZnONPs to glucosyltransferases and lactate dehydrogenase, enzymes critical for bacterial virulence and survival. Glucosyltransferases are responsible for synthesizing extracellular polysaccharides that form the structural matrix of the biofilm, while lactate dehydrogenase is involved in the metabolic pathways that produce lactic acid, contributing to the acidic environment that demineralizes tooth enamel [17-20].

The molecular docking results suggested that Luteo-ZnONPs inhibit enzyme activity, impairing bacterial metabolism and biofilm development. By binding to glucosyltransferases, Luteo-ZnONPs can inhibit the synthesis of the biofilm matrix, preventing the establishment and maintenance of biofilms. Binding to lactate dehydrogenase can disrupt the metabolic processes of the bacteria, reducing acid production and thereby mitigating enamel demineralization [21-22]. These interactions suggest that Luteo-ZnONPs not only exert direct antimicrobial effects but also interfere with critical bacterial functions necessary for biofilm formation and maintenance. This study highlights the synergistic effects of luteolin and Zn NPs, providing a foundation of developing advanced therapeutic strategies against dental caries. Combining the biofilm-targeting capabilities of luteolin with the antimicrobial properties of Zn NPs offers a promising alternative for preventing and treating dental caries [23-26]. The ability to target and disrupt biofilms while minimizing the risk of antibiotic resistance represents a significant advancement in the field of dental health. Specifically, the study aims to assess the ability of Luteo-ZnONPs to inhibit biofilm formation and reduce microbial viability. By combining the biofilm-targeting capabilities of luteolin with the antimicrobial properties of Zn NPs, we seek to develop amore effective strategy for preventing and treating dental caries. This study holds a significant implications for dental health, particularly in the context of developing new preventive and therapeutic strategies for dental caries [13]. Current treatments often fall short in effectively targeting biofilms and are increasingly limited by the growing issue of antibiotic resistance. Luteo-ZnONPs offer a promising alternative, providing a multifaceted approach to combating cariogenic microorganisms. By enhancing the targeting and antimicrobial efficiency of Luteo-ZnONPs could lead to more effective treatments that address the limitations of current therapies and reduce the incidence and severity of dental caries. In conclusion, the synthesis, characterization and evaluation of Luteo-ZnONPs represent a novel approach to tackling dental caries. Through both experimental and computational analyses, this study aims to demonstrate the potential of Luteo-ZnONPs as a powerful antimicrobial agent capable of disturbing biofilms and inhibiting cariogenic bacteria [14]. Luteo-ZnONPs enhance the bioactivity and efficacy against cariogenic microorganisms. Key measurable objectives of the present study include the synthesizing and assessing the nanoparticles' antimicrobial activity through minimum inhibitory concentration (MIC), evaluating its growth inhibitory activity using agar well plate method. Further, the present study aimed to analyze the binding potential between luteolin and *E. coli *gyrase B. This research not only contributes to the understanding of Luteo-ZnONPs' mechanism of action but also lays the groundwork for future developments in dental caries and improve oral health outcomes globally.

## Materials and methods

Synthesis of Luteo-ZnONPs 

Luteolin was purchased from Merck (India). The Luteo-ZnONPs were prepared by adopting the previous method with a slight modification [1-3]. To prepare the luteolin-ZnO nanodispersions, a stock solution was created by dissolving 0.5 g of luteolin in 200 mL of dimethylsulfoxide (DMSO, 10%) with heating for about one hour. After allowing the solution to settle, the supernatant was collected. Then, the Zn ion solution was prepared by dissolving 0.1 mM zinc nitrate (Zn(NO_3_)_2_) in deionized water. Separately, a sodium borohydride solution (1%) was prepared. These solutions were mixed under constant stirring to ensure thorough homogenization. A freshly prepared 0.1 M sodium borohydride solution was then added dropwise to the mixture while vigorously stirring to initiate the reduction of Zn ions, leading to the formation of Luteo-ZnONPs. The resulting NP solutions were centrifuged at 10,000 rpm for 20 minutes to separate Luteo-ZnONPs from any unreached materials and by-products. After discarding the supernatant, the NPs were washed multiple times with deionized water to eliminate residual reactants, ensuring the purity and stability of the synthesized Luteo-ZnONPs [1-3].

Characterization of Luteo-ZnONPs

Following the synthesis of Luteo-ZnONPs, characterization was conducted using several analytical techniques. UV-Vis spectroscopy (UV-1800-Shimadzu) was utilized to scan the NPs, detecting absorbance changes within the wavelength range of 200-700 nm. The particle size of Luteo-ZnONPs was calculated using the Debye-Scherrer equation, where λ represents the X-ray wavelength, β is the full width at half maximum (FWHM), and θ is the Bragg’s angle. FTIR using KBr pellets in the 500-4,000 cm⁻¹ range was employed to identify the functional groups present in the luteolin compound responsible for reducing Zn ions to NPs. These characterization techniques collectively provided comprehensive insights into the structural, morphological, and chemical properties of Luteo-ZnONPs [4-7,23].

Evaluation of antimicrobial efficacy by antimicrobial assay

Using a disc diffusion assay, the antimicrobial efficacy of Luteo-ZnONPs was assessed against *P. aeruginosa, S. mutans, E. faecalis, E. coli, *and *S. aureus *bacterial and fungal strains. Bacterial strains were cultured in lysogeny broth (LB) at 37°C for 24 hours and then spread onto LB agar plates to obtain bacterial suspensions. Fungi were cultured on potato dextrose agar at 25°C in darkness. Suspensions containing approximately 1 × 10^6^ colony-forming units (CFU) of each microorganism were spread on LB or potato dextrose (PD) agar plates using a sterilized glass spreader. Sterile filter paper discs (6 mm diameter) were loaded with fixed concentrations of Luteo-ZnONPs, while sterile water served as the negative control and standard antibiotics as positive controls. Plates were then incubated at 37°C for 24 hours. Following incubation, the diameter of the inhibitory zones (zone of inhibition, ZOI) formed around the discs loaded with different concentrations of Luteo-ZnONPs was measured to evaluate their antimicrobial activity. All experiments were conducted in triplicate to ensure the reliability and reproducibility of the results [12-16]. ZOI was represented as mean ± standard deviation. One-way analysis of variance (ANOVA) was performed to find the significance (p < 0.05) between the groups.

Cytotoxicity assay

The cytotoxic effects of Luteo-ZnONPs on MG63 cells were assessed using the 3-4, 5-dimethylthiazolyl-2)-2, 5-diphenyltetrazoliumbromide (MTT) assay. MG63 cells were seeded in a 86-well plate at a density of 1 x 10^4^ cells/well and allowed to adhere overnight at 37^o^C in a humified atmosphere containing 5% CO_^2^_. Doxorubicin was used as the positive control. The cells were treated with varying concentration (0, 10, 25, 50, 75, and 100 µg/mL, respectively) of the doxorubicin and Luteo-ZnONPs for 24 hours. Following treatment, 20 µL of MTT solution (5 mg/ mL in phosphate buffer saline) was added to each well and incubated for four hours at 37^o^C. The experiment was done in triplicates, and the results were represented as mean ± standard deviation. The Wilcoxon test was used to analyze the statistical significance between the groups (p < 0.05).

Molecular docking studies

A molecular docking study employing the AutoDock method [5] was conducted to investigate the interaction between Luteo-ZnONPs and their protein receptor gyrase B on *E. coli*, extracted from the RCBS Protein Data Bank (PDB: 6F86). Gyrase B is an essential enzyme in *E. coli *that plays a key role in DNA replication by introducing negative supercoils into DNA strands. The crystallographic information file (CIF) of Luteo-ZnONPs was obtained and converted into PDB format to serve as a ligand in the docking stimulations. Prior to initiating the stimulations, Luteo-ZnONPs and gyrase B receptor were prepared by assigning Gasteiger partial charges, Kolman charges, and adding polar hydrogen atoms. The Lamarckian genetic algorithm was employed for the docking process. Autogrid parameters were adjusted to generate a comprehensive grid map covering the entire surface of the 6F86 protein. The docking stimulations aimed to identify the optimal binding mode and binding sites of Luteo-ZnONPs with 6F86. The pose with the most negative binding energy was selected as the best docked model, which was subsequently analyzed to visualize the binding interaction and sites using BIOVIA software [5]. This approach provided insights into how Luteo-ZnONPs interact with 6F86, potentially affecting bacterial fatty acid metabolism.

## Results

UV-vis spectroscopy analysis 

**Figure 1:**
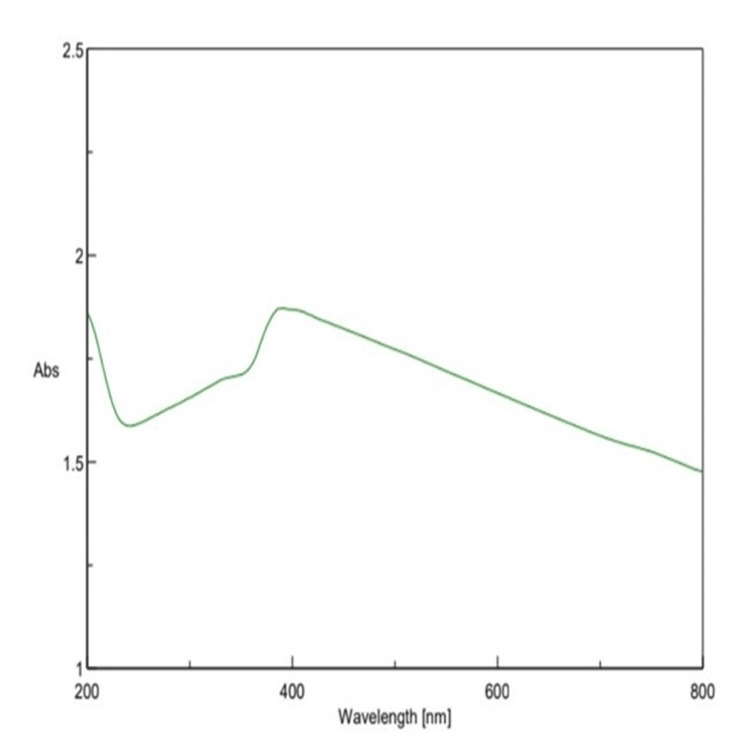
UV-vis absorption spectra of luteolin-loaded zinc oxide nanoparticles UV-vis: Ultraviolet-visible

Characterization of Luteo-ZnONPs using UV-vis spectroscopy revealed a distinct exciton band at 377 nm. This absorption peak closely mirrors the bulk exciton absorption of Luteo-ZnONPs, indicating the formation of special Luteo-ZnONPs with an average size range of 40-60 nm. The rapid increase in absorbance upon excitation from the NPs' ground state to its excited state further confirms their optical properties. However, a subsequent decrease in radiation absorption suggests some degree of nanoparticle agglomeration post-synthesis. The bandgap energy of Luteo-ZnONPs was calculated to be 3.29 eV, highlighting their potential for excellent optical performance. These findings underscore the successful synthesis of Luteo-ZnONPs and their promising optical characteristics suitable for diverse applications (Figure 1).

FTIR analysis

**Figure 2:**
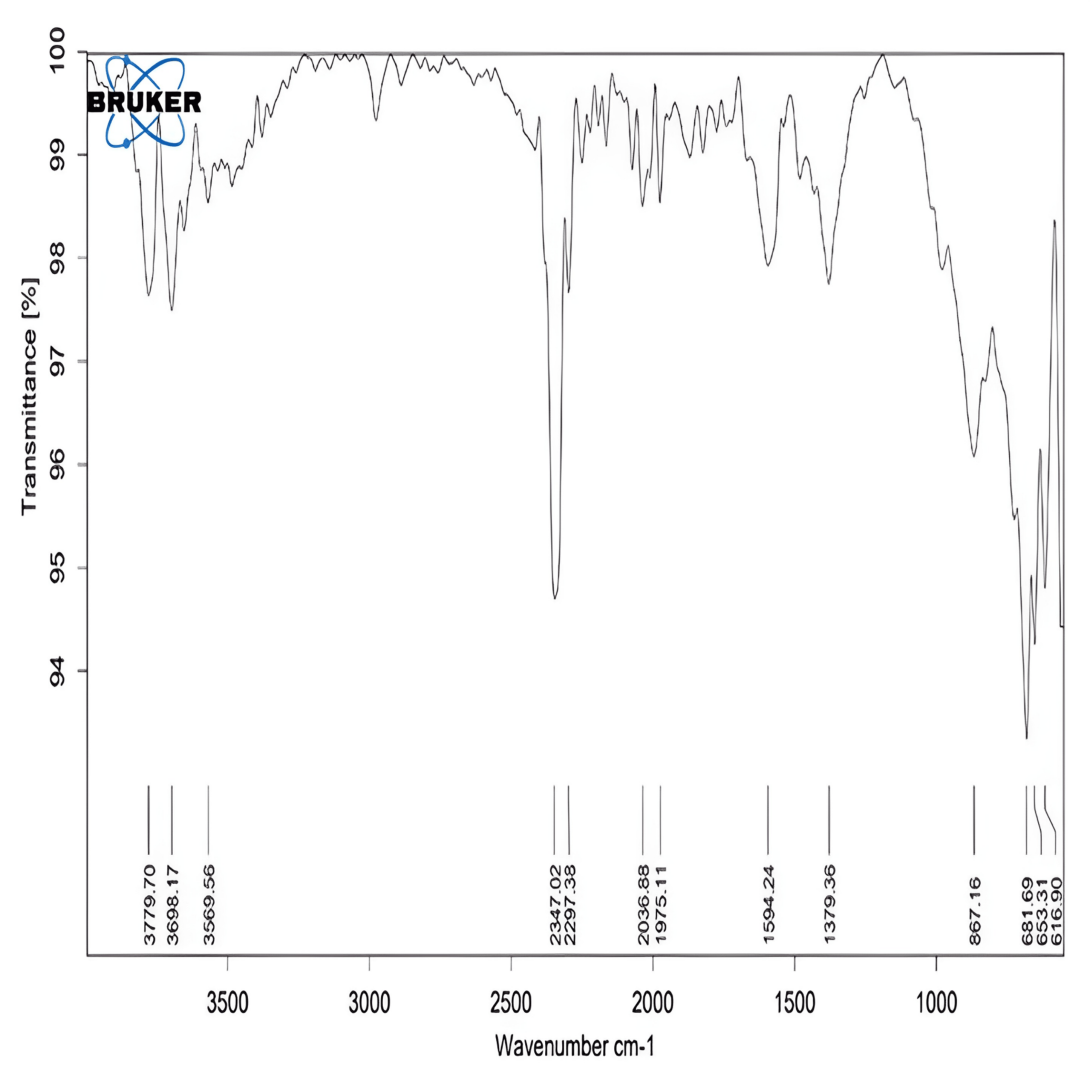
FTIR spectra of luteolin-loaded zinc oxide nanoparticles FTIR: Fourier transform infrared

FTIR analysis of Luteo-ZnONPs was employed to confirm the presence of functional groups from the luteolin compound involved in the reduction of Zn^2^+ to Zn^0^ and in the capping and stabilization of bio-reduced Luteo-ZnONPs. Figure 2 of the IR spectrum shows a broad peak at 3,371 cm^-1^, predominantly attributed to the O-H stretching vibration of alcohol functionalities, indicating the involvement of bioactive compounds with OH groups in the formation of Luteo-ZnONPs. Additionally, a weaker broad peak around 3,400 cm^-1^ in the Luteo-ZnONPs' IR spectrum compared to the extract suggests the presence of these bioactive compounds. Other notable peaks observed at 2,890 cm^-1^ and a slightly split peak at 1,639 cm^-1^ correspond to C-H stretching vibrations of alkane groups and ketones, respectively. The significant peak at approximately 499 cm^-1^ in the Luteo-ZnONPs' FTIR spectrum, indicative of metal-oxygen (M-O) bonds, further supports the formation of NPs. Analysis of the luteolin extract's spectrum revealed the potential involvement of phytochemicals such as phenols, terpenes, and flavonoids in the reduction of metal ions to Luteo-ZnONPs.

Antimicrobial potential of Luteo-ZnONPs

**Figure 3:**
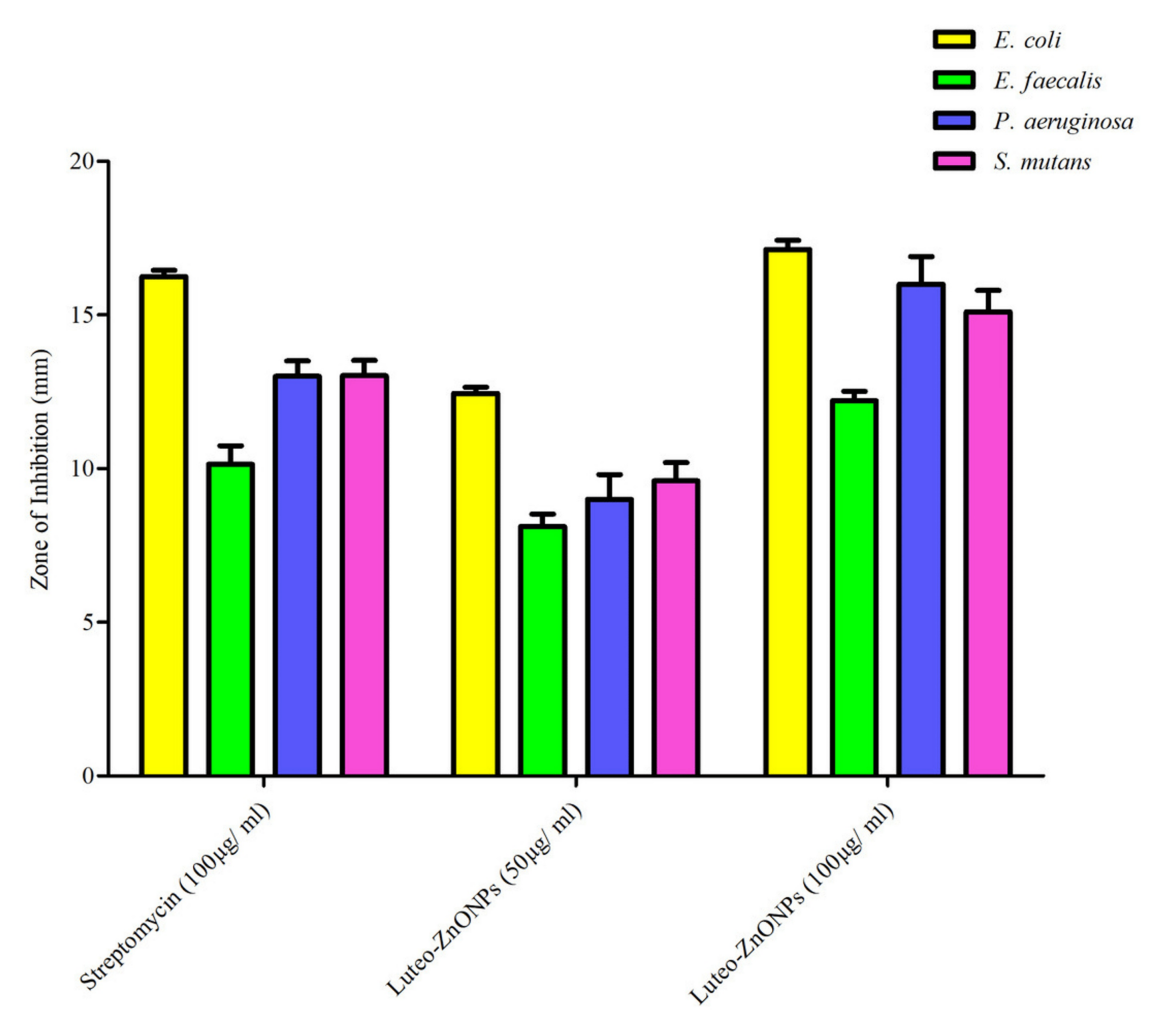
Antimicrobial activity of Luteo-ZnONPs against different pathogens Luteo-ZnONPs: Luteolin-loaded zinc oxide nanoparticles Image credit: Meenakshi Sundaram

**Figure 4:**
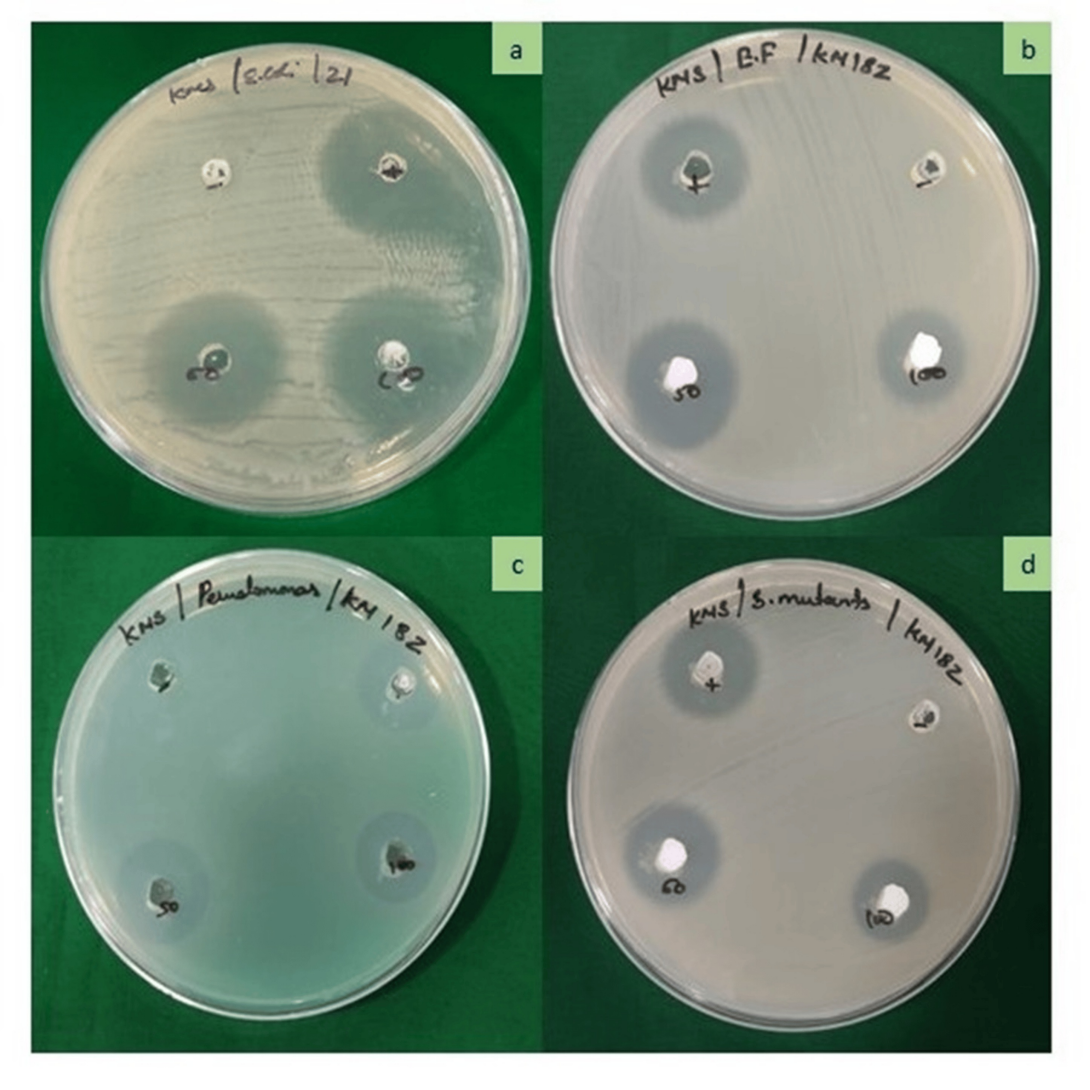
Antimicrobial activity of luteolin-loaded zinc oxide nanoparticles for bacterial and fungal strains. (a) Escherichia coli, (b) Enterococcus faecalis, (c) Pseudomonas aeroginosa, (d) Streptococcus mutans Image credit: Meenakshi Sundaram

The antimicrobial efficacy of Luteo-ZnONPs was evaluated against four bacterial strains: *E. coli, E. faecalis, P. aeruginosa, *and *S. mutans *(Table 1)*. *Streptomycin (100 µg/ml) shows varying levels of inhibition, with the largest ZOI against *E. coli *at 16.25 mm and the smallest against *E. faecalis* at 10.14 mm. Its effectiveness against *P. aeruginosa* and *S. mutans* is similar, with inhibition zones of 13.56 mm and 13.02 mm, respectively. In comparison, Luteo-ZnONPs at 50 µg/ml exhibit lower antibacterial activity, with the largest ZOI against *E. coli* (12.45 mm) and the smallest against *E. faecalis* (8.12 mm). When the concentration of Luteo-ZnONPs is increased to 100 µg/ml, their effectiveness improves, showing the largest inhibition zone against *E. coli* (17.13 mm) and notably activity the other bacteria as well, indicating enhanced antibacterial potential with higher concentrations (Figures 3, 4).

**Table 1:** Results of one-way ANOVA ANOVA: Analysis of variance

ANOVA: single factor				
Groups	Count	Sum	Average	Variance
Escherichia coli	3	45.83	15.27667	6.186133
Enterococcus faecalis	3	30.47	10.15667	4.182233
Pseudomonas aeruginosa	3	39.36	13.12	11.8416
Streptococcus mutans	3	37.72	12.57333	7.712133

ANOVA						
Source of variation	SS	df	MS	F	P-value	F crit
Between groups	39.82057	3	13.27352	1.774411	0.229649	4.066181
Within groups	59.8442	8	7.480525			
Total	99.66477	11				

The ANOVA test showed that there was a significant difference between the groups at a 5% significance level. The F- value was 4.5594 with a corresponding p-value of 0.033. Since the p-value was lesser than 0.05, it indicated that there was a statistically significant difference between the means of the three groups (streptomycin, Luteo-ZnONPs 50 and 100 µg/ml respectively). The F critical value (F crit) was 3.885, and the calculated F-value (4.594) was greater than the critical value, further supporting that the differences between groups were significant. 

Cytotoxicity analysis

**Figure 5:**
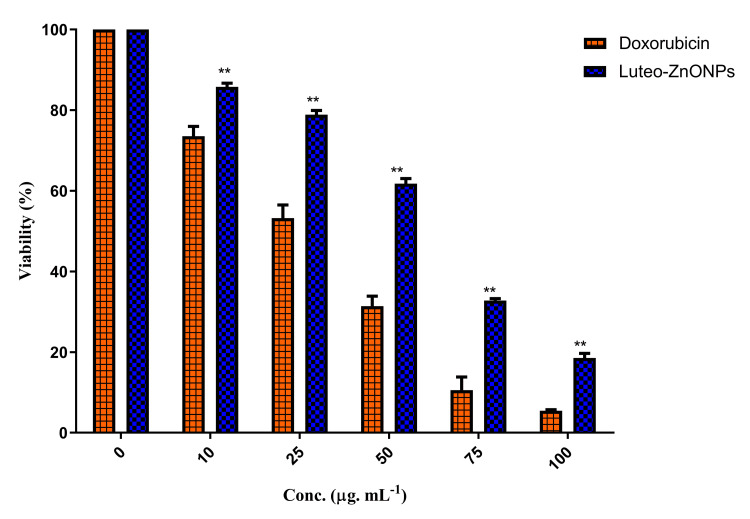
Cytotoxicity of Luteo-ZnONPs against MG63 cells using the MTT assay Luteo-ZnoNPs: Luteolin zinc oxide nanoparticles; MTT: 3-4, 5-dimethylthiazolyl-2)-2, 5-diphenyltetrazoliumbromide Doxorubicin was used as positive control. Doxorubicin and Luteo-ZnoNPs showed a concentration-dependent cytotoxicity on MG63, a osteosarcoma cell lines. The results were represented as mean ± standard deviation.  Wilcoxon matched paired test revealed that the treatments were statistically significant (p < 0.0014, indicated as **)

The MTT assay revealed a dose-dependent reduction in cell viability in MG63 cells upon treatment with both doxorubicin and Luteo-ZnONPs (Figure 5). Doxorubicin, the positive control showed a concentration dependent viability decrease compared to the control. Similarly, Luteo-ZnONPs showed a gradual decrease in cell viability with increasing the concentrations as starting from 85%, 78.9%, 61.8%, 32.78%, and 18,75% on 0, 10, 25, 50, and 100 µg/mL, respectively. The statistical analysis using the Wilcoxon matched-pair test indicated that the reduction in cell viability between doxorubicin and Luteo-ZnONPs was statistically significant, with a p-value of 0.0014, confirming that both treatments significantly reduce cell viability on concentration dependently. Meanwhile, the MTT assay also showed that Luteo-ZnONPs had lesser toxicity than doxorubicin.

Molecular docking analysis

**Figure 6:**
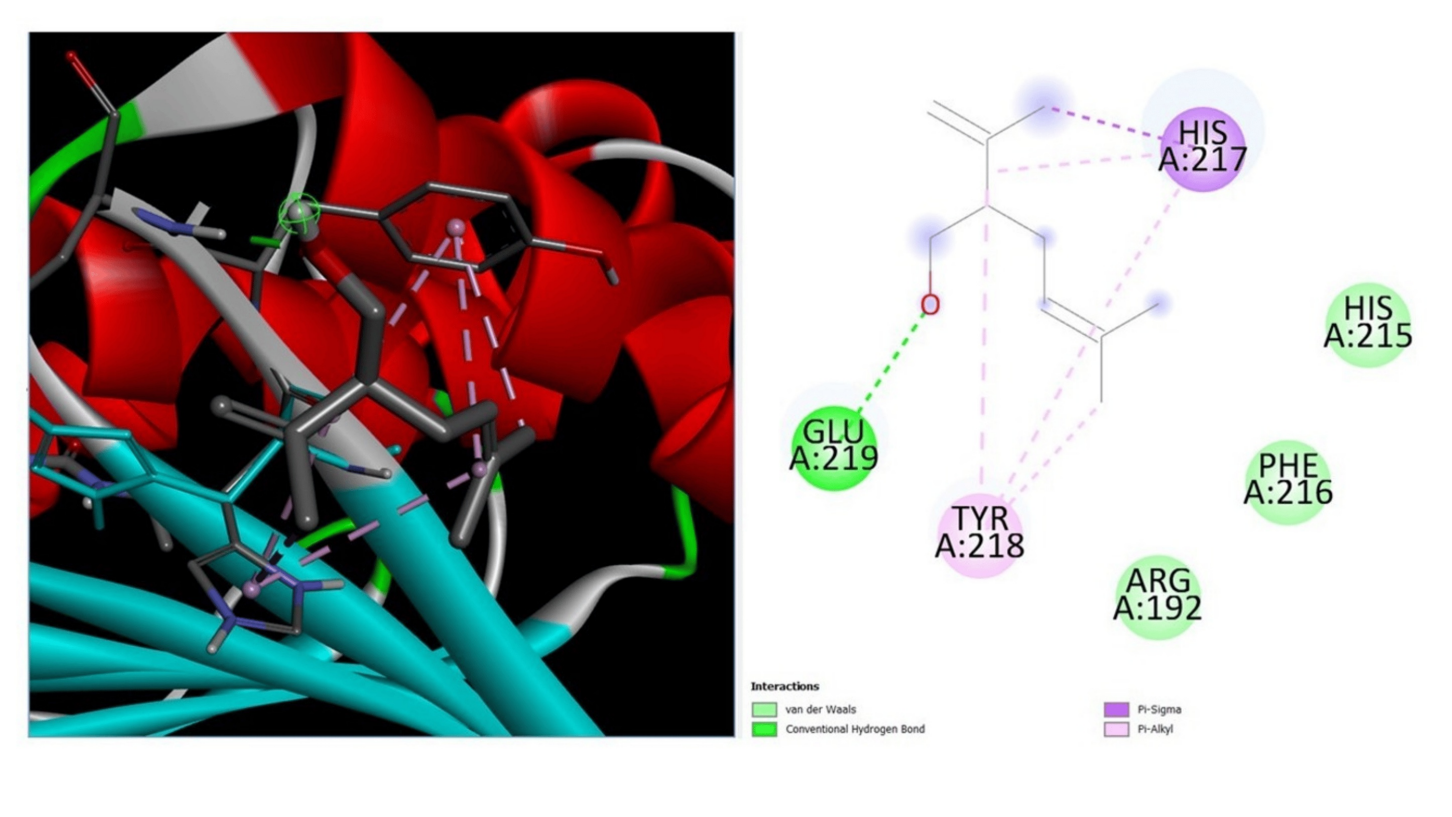
Molecular docking study of receptor, ligand best docking pose, and various luteolin-loaded zinc oxide nanoparticles interactions with amino acids contribute to cavity formation Image credit: Meenakshi Sundaram

A catalytic trial tunnel comprising glutamic Acid (GLU) (219), histidine (HIS) (217), and tyrosine (TYR) (218) is located within the active site of gyrase B on *E. coli* (PDB : 6F86), where Luteo-ZnONPs have the potential to significantly modulate, inhibit, or even disrupt the enzyme's catalytic activity. Furthermore, the conserved active site residues of the Luteo-ZnONPs receptor across Gram-positive and Gram-negative bacteria designate the 6F86 protein as an appealing therapeutic target. This suggests its potential for developing innovative and broad-spectrum antimicrobial drugs as selective and nontoxic Luteo-ZnONPs inhibitors. In order to predict the in vitro efficacy of Luteo-ZnONPs, molecular docking studies using the ligand-6F86 model were conducted. This investigation aimed to elucidate the optimal orientation of NPs within the receptor and to uncover critical noncovalent interactions between the active site of the receptor and Luteo-ZnONPs. Such insights could pave the way for the development of novel drugs and further biological research (Figure 6).

## Discussion

The discussion focuses on the potential of Luteo-ZnONPs as a novel approach to combat dental caries, considering their antimicrobial properties and mechanism of action against cariogenic bacteria. Dental caries, primarily caused by the acid-producing activities of cariogenic microorganisms like* Streptococcus mutans* and *Enterococcus faecalis*, poses a significant global health challenge [12-14]. Traditional treatments are often inadequate due to biofilm resilience and emerging antibiotic resistance issues, necessitating innovative therapeutic strategies [1]. NPs have been extensively researched for their antimicrobial effects, including disruption of microbial cell membranes and interference with microbial functions [12-16]. However, the study explores Luteo-ZnONPs as an alternative, leveraging lavender's known antimicrobial benefits and Zn's antibacterial properties. Luteo-ZnONPs were synthesized and characterized, confirming their size, shape, and stability and were subsequently evaluated for their antibacterial efficacy against cariogenic bacteria through various assays. The results indicate that Luteo-ZnONPs effectively inhibit the growth of cariogenic bacteria, suggesting their potential as a therapeutic agent for preventing dental caries. Although the doxorubicin, a well-established therapeutic agent, exhibited a more pronounced cytotoxic effect, Luteo-ZnONPs demonstrated a considerable potential in reducing cell viability. This suggested that it might possessed significant anticancer properties, possibly offering an alternative or complementary approach to conventional therapies. The statistical significance of the results also supported that Luteo-ZnONPs had lesser cytotoxicity than the doxorubicin. Previously, Mishra et al. (2017) also showed that the biosynthesized ZnONPs were lesser toxic than doxorubicin [27].

Molecular docking studies further revealed strong binding affinities between Luteo-ZnONPs and key bacterial enzymes involved in biofilm formation and acid production, respectively. By inhibiting these enzymes, Luteo-ZnONPs disrupt bacterial metabolism and biofilm development, thereby potentially reducing the progression of dental caries [1-5]. This dual mechanism, direct antimicrobial activity and inhibition of virulence factors, underscore the potential of Luteo-ZnONPs as a multifaceted approach to caries prevention [1]. Furthermore, the use of Luteo-ZnONPs presents advantages over AgNPs, such as potentially lower cytotoxicity and environmental impact, which are significant considerations for clinical and ecological applications [17-20]. The integration of compounds like luteolin with essential trace elements like Zn aligns with trends toward sustainable and biocompatible antimicrobial solutions in dentistry. Further research is essential to fully realize the potential of Luteo-ZnONPs in dental care. Optimal dosages and application methods need to be thoroughly investigated to ensure that Luteo-ZnONPs provide maximum therapeutic benefits while minimizing any adverse effects. This included determining the appropriate concentrations for effective antibacterial action without causing harm to oral tissues. Additionally, it is crucial to explore the impact of Luteo-ZnONPs on the overall balance of the oral microbiome. Ensuring that their use does not disrupt beneficial microbial communities is vital for maintaining oral health and preventing unintended consequences. Long-term studies should also be conducted to evaluate the effects of Luteo-ZnONPs on dental tissues and their potential interactions with existing dental materials. This is important to confirm their safety and compatibility in real-world scenarios, where they might be used in conjugation with other dental treatments and materials. Assessing the durability and performance of Luteo-ZnONPs over extended periods will help establish their practical viability in clinical applications [27-28].

Despite the promising findings, several limitations must be addressed before Luteo-ZnONPs can be widely adopted. One significant challenge is the scalability of the synthesis process. The transition from laboratory-scale production to large-scale manufacturing could present difficulties, including increased costs and technical complexities. Furthermore, the environmental impact of NP disposal needs careful consideration. The potential accumulation of NPs in the environment and their long-term effects on ecosystems must be evaluated to ensure sustainable use [29]. 

Individual variability in response to NP-based treatments is another concern. Personalized approaches may be required to tailor treatments to specific patient needs and ensure efficacy across diverse populations. Comprehensive clinical trials will be crucial to assessing the overall effectiveness, safety, and potential side effects of Luteo-ZnONPs. Regulatory assessments will also be necessary to meet the required standards for approval and ensure that these NPs are a viable and safe option for caries prevention and treatment. Hence, the safety, stability, and storage assessment is required before further processing the Luteo-ZnONPs. Addressing these limitations through rigorous research and regulatory scrutiny will be key to translating laboratory successes into effective, real-world dental care solutions.

## Conclusions

In conclusion, Luteo-ZnONPs demonstrate promising antimicrobial efficacy against cariogenic bacteria, highlighting their potential as an innovative strategy for preventing and treating dental caries. Through their ability to inhibit key enzymes involved in biofilm formation and acid production, Luteo-ZnONPs offer a dual mechanism to disrupt bacterial virulence and mitigate enamel demineralization. Further research and clinical trials are warranted to validate their effectiveness, safety, and feasibility as a therapeutic agent in dental care. Luteo-ZnONPs represent a step forward in addressing the challenges posed by dental caries, offering a biocompatible and potentially sustainable alternative to conventional antimicrobial treatments.
